# Changing Etiological Patterns of Pediatric Meningitis: A Three-Year Experience from a Tertiary Pediatric Emergency Hospital in Romania

**DOI:** 10.3390/medicina62081497

**Published:** 2026-08-04

**Authors:** Dan Dumitru Vulcanescu, Florin George Horhat, Cristina Ciortuz, Norberth Istvan Varga, Iulia Cristina Bagiu

**Affiliations:** 1 Department of Microbiology, “Victor Babes” University of Medicine and Pharmacy, Eftimie Murgu Square No. 2, 300041 Timisoara, Romania; dan.vulcanescu@umft.ro (D.D.V.); bagiu.iulia@umft.ro (I.C.B.); 2 Multidisciplinary Research Center on Antimicrobial Resistance (MULTI-REZ), Microbiology Department, “Victor Babes” University of Medicine and Pharmacy, Eftimie Murgu Square No. 2, 300041 Timisoara, Romania; 3 Clinical Laboratory, “Louis Țurcanu” Emergency Clinical Hospital for Children, 300011 Timișoara, Romania; cristina.ciortuz@umft.ro; 4 Doctoral School, “Victor Babeș” University of Medicine and Pharmacy, 300041 Timișoara, Romania; norberth.varga@umft.ro; 5 Department of Nursing, “Victor Babes” University of Medicine and Pharmacy, Eftimie Murgu Square, No. 2, 300041 Timisoara, Romania; 6 Department of Infectious Diseases, “Louis Țurcanu” Emergency Clinical Hospital for Children, 300011 Timișoara, Romania

**Keywords:** pediatric meningitis, viral meningitis, bacterial meningitis, enterovirus, cerebrospinal fluid, molecular diagnostics, etiological surveillance, children

## Abstract

*Background and Objectives*: Pediatric meningitis remains a major diagnostic and therapeutic emergency because clinical presentation may be nonspecific and early etiological distinction directly influences treatment, infection control, and epidemiological surveillance. Although bacterial meningitis has traditionally been emphasized because of its severity, the wider use of rapid molecular methods has increased the detection of viral etiologies. This study aimed to evaluate the etiological distribution of confirmed pediatric meningitis episodes and to assess temporal, age-related, and sex-associated patterns, with particular attention to the observed shift from bacterial toward viral meningitis. *Materials and Methods*: A retrospective single-center analysis was performed using microbiological data from suspected meningitis episodes investigated between 2023 and 2025 at the “Louis Țurcanu” Emergency Hospital for Children, Timișoara. Confirmed episodes were classified as bacterial, viral, or fungal according to the identified etiological agent. The distribution of suspected and confirmed episodes, annual etiological patterns, age-group differences, sex-associated variation, and identified pathogens were assessed using descriptive statistics and trend analysis. *Results*: Among 641 suspected episodes, 58 were microbiologically confirmed, corresponding to an overall positivity rate of 9.05%. Viral meningitis was the most frequent etiology, accounting for 30 episodes (51.72%), followed by bacterial meningitis with 22 episodes (37.93%) and fungal meningitis with 6 episodes (10.34%). Etiological distribution changed significantly over the study period: viral episodes increased from 22.22% in 2023 to 77.78% in 2025, while bacterial episodes decreased from 66.67% to 22.22%. Viral meningitis predominated in children aged 1–6 years and was more frequent among male patients, whereas bacterial meningitis remained more frequent in infants. Enterovirus became the dominant identified agent in 2025. *Conclusions*: The local epidemiology of confirmed pediatric meningitis showed an observed redistribution among confirmed episodes toward viral etiologies, particularly enteroviral meningitis, alongside a proportional decline in bacterial episodes. These findings support continued etiological surveillance and the integration of rapid molecular testing with conventional microbiology in the diagnostic management of suspected pediatric meningitis.

## 1. Introduction

Meningitis in children remains an important clinical, microbiological, and public health concern because of its potentially severe evolution, the need for rapid therapeutic decisions, and the possible epidemiological implications of specific etiologies. Although the overall incidence of pediatric meningitis is relatively low, delayed recognition or inappropriate initial management may be associated with significant morbidity, neurological sequelae, and mortality. For this reason, suspected meningitis continues to be managed as a medical emergency, requiring close collaboration between clinicians, microbiology laboratories, and public health services [1,2].

Traditionally, bacterial meningitis has represented the main diagnostic priority in children because of its rapid progression and the need for immediate antimicrobial treatment. The major bacterial pathogens vary according to age, vaccination status, and clinical context, with particular relevance of *Streptococcus pneumoniae*, *Neisseria meningitidis*, *Haemophilus influenzae*, and neonatal pathogens such as group B streptococci and Gram-negative bacilli. In addition, some bacterial etiologies, particularly meningococcal disease, require urgent reporting, epidemiological investigation, and prophylaxis of close contacts [3,4,5].

However, the etiological landscape of pediatric meningitis extends beyond the classical bacterial pathogens. It includes a broad spectrum of viral, bacterial, fungal, and other less frequent causes, whose distribution may vary according to age, immune status, vaccination background, seasonality, and diagnostic availability. While bacterial meningitis has been extensively studied because of its severity and urgent therapeutic implications, non-bacterial etiologies, particularly viral meningitis, remain comparatively less characterized in children [6]. The implementation of vaccination programs has reduced the burden of several classical bacterial pathogens, while the wider availability of molecular diagnostic methods has improved the detection of viral agents [7]. Viral meningitis, especially enteroviral meningitis, is therefore increasingly recognized as an important cause of confirmed pediatric meningitis. Although it is generally associated with a more favorable prognosis than bacterial disease, it remains clinically relevant because it may mimic bacterial meningitis at presentation, lead to hospitalization and empirical antimicrobial use, and require rapid etiological clarification [8].

Cerebrospinal fluid analysis remains the diagnostic framework for suspected meningitis. Conventional methods, including cytology, biochemistry, Gram stain, culture, and antimicrobial susceptibility testing, remain essential for defining the inflammatory profile and for confirming bacterial or fungal etiologies. However, the etiological confirmation of viral meningitis relies mainly on molecular methods, since viral agents are not detected by Gram stain and are not routinely identified by conventional culture workflows. Rapid PCR-based assays therefore complement, rather than replace, classical microbiological methods, providing early viral detection, supporting antimicrobial de-escalation, and improving clinical and epidemiological interpretation [1,9]. Local epidemiological data are particularly important because the distribution of meningitis etiologies may differ according to patient age, referral patterns, vaccination coverage, seasonality, and diagnostic availability. Local surveillance can reveal shifts in pediatric meningitis etiology and guide diagnostic workflows. The increasing proportion of viral episodes supports renewed attention to molecular testing in suspected meningitis.

Etiological patterns also differ according to clinical setting. Community-acquired meningitis is shaped mainly by age and vaccination status, whereas neonatal meningitis is more closely associated with prematurity, perinatal factors, and neonatal pathogens. In hospitalized or critically ill children, healthcare-associated meningitis may also be influenced by neurosurgical procedures, invasive devices, prolonged hospitalization, and immune status. These distinctions are important when interpreting microbiological findings across pediatric age groups and hospital departments [3,4,6].

The aim of this study was to evaluate the etiological distribution of confirmed pediatric meningitis episodes diagnosed in a tertiary emergency children’s hospital between 2023 and 2025. The analysis focused on temporal changes in bacterial, viral, and fungal etiologies, as well as age-group, sex-associated, and pathogen-level distribution.

## 2. Materials and Methods

### 2.1. Study Design

A retrospective, single-center, laboratory-based study was conducted at the Emergency Hospital for Children “Louis Țurcanu”, Timișoara, Romania, a tertiary pediatric hospital and major regional referral center in western Romania. The study included hospitalized pediatric patients aged 0–18 years who were investigated for suspected meningitis between January 2023 and December 2025 and had cerebrospinal fluid submitted to the microbiology laboratory.

The unit of analysis was the suspected meningitis episode, defined as a hospitalization during which CSF microbiological investigation was requested for possible meningitis. Multiple CSF samples collected during the same hospitalization or clinical episode were counted once. A subsequent hospitalization separated in time was considered a distinct episode, even when it involved the same patient. All eligible suspected episodes were included in the initial analysis and classified as microbiologically negative or confirmed according to CSF results. Confirmed episodes were defined by the identification of a clinically relevant bacterial, viral, or fungal pathogen in CSF through culture-based and/or molecular methods within the routine clinical and laboratory diagnostic workflow. Episodes with insufficient data for etiological classification were excluded. Patients older than 18 years, non-hospitalized patients, episodes without CSF microbiological testing, and episodes with insufficient data for etiological classification were excluded. When multiple CSF samples were collected during the same clinical episode, the episode was counted once to avoid duplication.

The extracted variables included year of diagnosis, age group, sex, area of origin, requesting or admitting ward, microbiological result, identified etiological agent or agent group, etiological category, and method of etiological confirmation. Area of origin was recorded according to the available hospital data. The method of confirmation was classified as culture-based, molecular, or both, when applicable.

### 2.2. Methodological Workflows

Cerebrospinal fluid was handled as an urgent biological sample. On arrival in the laboratory, the sample was assessed macroscopically and processed according to test priority and available volume. The diagnostic workflow included conventional microbiological methods and molecular testing, depending on clinical indication, sample availability, and laboratory workflow. Conventional cerebrospinal fluid investigation included direct Gram staining, bacterial and fungal culture, and subsequent identification and antimicrobial susceptibility testing when growth was obtained. Gram staining was performed using the routine laboratory staining kit according to standard procedures.

Culture was performed using both manual kflows during the study period. For culture-based diagnosis, CSF was inoculated primarily onto blood agar and chocolate agar and incubated at 35–37 °C in a 5% CO_2_ atmosphere. Additional media, such as MacConkey agar or enrichment broth, were used when clinically indicated or according to routine laboratory protocols. Depending on the period and workflow, culture plates were processed manually or by automated microbiology systems, including WASPLab-based processing (COPAN, Brescia, Italy), incubation, digital imaging, and plate reading support.

Positive cultures were further processed for microorganism identification and antimicrobial susceptibility testing. Identification was performed using the I-dOne system (Alifax S.r.l., Polverara, Italy) and/or MALDI-TOF mass spectrometry (Bruker Daltonics, Bremen, Germany), depenMALDI Biotyper sirius system and availability. For fungal investigation, CSF was inoculated onto Sabouraud dextrose agar, either manually or through WASPLab-based processing, depending on the laboratory workflow. Positive fungal cultures were identified using the same platforms described for bacterial isolates, including the I-dOne system and/or MALDI-TOF mass spectrometry. Antimicrobial and antifungal susceptibility testing was performed as part of the routine laboratory workflow; however, isolate-level susceptibility results were outside the scope of the present study and were not included in the analysis.

Molecular testing was performed using the BioFire FilmArray Meningitis/Encephalitis Panel (BioFire Diagnostics, Salt Lake City, UT, USA; bioMérieux), according to the manufacturer’s instructions. The assay was used for rapid multiplex detection of bacterial, viral, and yeast pathogens directly from cerebrospinal fluid.

### 2.3. Ethical Considerations and Statistical Analysis

The study was conducted in accordance with the Declaration of Helsinki and was part of a series of retrospective research studies approved by the Ethics Committee of the Emergency Hospital for Children “Louis Țurcanu”, Timișoara, Romania, approval no. 44/2026. Written informed consent for hospitalization, diagnostic procedures, and the use of medical data for institutional purposes was obtained from the parent or legal guardian at admission, in accordance with hospital policy. Data used for this retrospective study were anonymized during extraction and analyzed in accordance with institutional data protection procedures.

Data management was performed using Microsoft Excel 2021, version 2605 (Microsoft Corporation, Redmond, WA, USA). Statistical analyses were performed using MedCalc Statistical Software, version 23.6.0 (MedCalc Software Ltd., Ostend, Belgium), and power analysis was performed using G*Power, version 3.1.9.7 (Heinrich Heine University Düsseldorf, Düsseldorf, Germany).

Descriptive statistics were used to summarize suspected and confirmed episodes, annual positivity rates, etiological distribution, age-group distribution, demographic and hospitalization characteristics and method of etiological confirmation. Categorical variables were reported as absolute numbers and percentages. Continuous variables, including age, were not normally distributed at the Shapiro–Wilk test and were reported as medians and interquartile ranges. Associations between categorical variables were assessed using the Chi-square test. The Fisher–Freeman–Halton exact test was additionally performed, where applicable. Temporal trends were evaluated using the Cochran–Armitage trend test. Age was compared between viral and non-viral confirmed episodes using the Mann–Whitney U test. Univariable binary logistic regression was used to assess age as a predictor of viral etiology, with results expressed as odds ratios and 95% confidence intervals. Statistical significance was set at *p* < 0.05.

Regarding sample size, for the main binary trend analyses, a Chi-square framework with 1 degree of freedom was used as an approximation of the Cochran–Armitage trend test. Assuming a large effect size (Cohen’s w = 0.50), α = 0.05, and power = 0.95, the minimum required sample size was 52 confirmed episodes, which was exceeded by the final dataset of 58 confirmed episodes. For the global Chi-square analysis of etiological distribution, the observed effect size was Cohen’s w = 0.60, with an estimated post hoc power of approximately 0.97 for 4 degrees of freedom.

## 3. Results

During the study period, 641 suspected pediatric meningitis episodes were investigated. Of these, 58 were microbiologically confirmed, corresponding to an overall positivity rate of 9.05%. As shown in Table 1, the number of suspected episodes increased from 160 in 2023 to 288 in 2025, while the annual positivity rate varied between 6.74% and 11.25%. No statistically significant difference in positivity rate was observed between years (χ^2^ = 2.23, df = 2, *p* = 0.327), and the Cochran–Armitage test did not identify a significant linear trend over time (Z = −0.44, *p* = 0.661).

Further demographic data for positive patients are presented in Table 2. The cohort included 58 microbiologically confirmed episodes, with a median age of 2 years (IQR: 0–4.75 years; range: 0–15 years). Most patients belonged to the 1–6 years age group (48.28%). Sex distribution was nearly balanced, with a slight male predominance (51.72%). Most patients were from urban areas (65.52%). The most frequent admitting departments were the Emergency Department (41.38%) and the ICU (32.8%).

Table 3 shows that among the 58 confirmed meningitis episodes, viral etiologies were the most frequent overall, accounting for 30 episodes (51.72%), followed by bacterial etiologies with 22 episodes (37.93%) and fungal etiologies with 6 episodes (10.34%). The etiological distribution changed significantly over the study period (χ^2^ = 20.91, df = 4, *p* < 0.001). Because of sparse expected cell counts, the analysis was additionally verified using the Fisher–Freeman–Halton exact test (*p* < 0.001).

Bacterial meningitis decreased from 66.67% of confirmed episodes in 2023 to 22.22% in 2025, showing a significant downward trend (Z = −2.94, *p* = 0.003). In contrast, viral meningitis increased from 22.22% in 2023 to 77.78% in 2025, with a significant upward trend (Z = 3.74, *p* < 0.001). No significant temporal trend was observed for fungal meningitis (Z = −1.46, *p* = 0.145).

Etiological distribution showed a significant association with sex (χ^2^ = 6.67, df = 2, *p* = 0.036). Because of sparse expected cell counts, the analysis was additionally verified using the Fisher–Freeman–Halton exact test (*p* = 0.042).

Among episodes involving male patients, 20 of 30 (66.67%) were viral, compared with 10 of 28 episodes (35.71%) involving female patients. In contrast, bacterial and fungal etiologies represented higher proportions of episodes involving female patients: 46.43% and 17.86%, respectively, compared with 30.00% and 3.33% among episodes involving male patients. All these data are detailed in Table 4.

Table 5 describes the relationship between etiology and age group. Etiological distribution differed significantly according to age group (χ^2^ = 21.44, df = 4, *p* < 0.001). Because of sparse expected cell counts, the analysis was additionally verified using the Fisher–Freeman–Halton exact test (*p* < 0.001).

In infants aged <1 year, bacterial meningitis was the predominant etiology, accounting for 13 of 21 confirmed episodes (61.90%), while viral meningitis represented only 3 episodes (14.29%). In contrast, viral meningitis predominated in children older than 1 year, accounting for 22 of 28 episodes (78.57%) in the 1–6-year group and 5 of 9 episodes (55.56%) in the 7–18-year group. Overall, among children older than 1 year, viral meningitis accounted for 27 of 37 confirmed episodes (72.97%). A significant increasing trend was observed for viral etiologies across age groups (Z = 3.10, *p* = 0.002), while bacterial etiologies showed a decreasing trend (Z = −2.12, *p* = 0.034).

Viral meningitis was observed in older children, with a median age of 4 years (IQR: 2–5), whereas bacterial and fungal episodes had median ages of 0 years. In an exploratory binary comparison using the Mann–Whitney U test, viral episodes had a significantly higher median age than non-viral episodes (4 years [IQR: 2–5] versus 0 years [IQR: 0–2], *p* < 0.001). Univariable logistic regression also suggested an association between increasing age and viral etiology, with each additional year of age associated with higher odds of viral meningitis (OR = 1.22 per year, 95% CI: 1.01–1.48, *p* = 0.041). This finding should be interpreted cautiously given the limited sample size and the recording of age in completed years. This is shown in Table 6.

**Figure 1:**
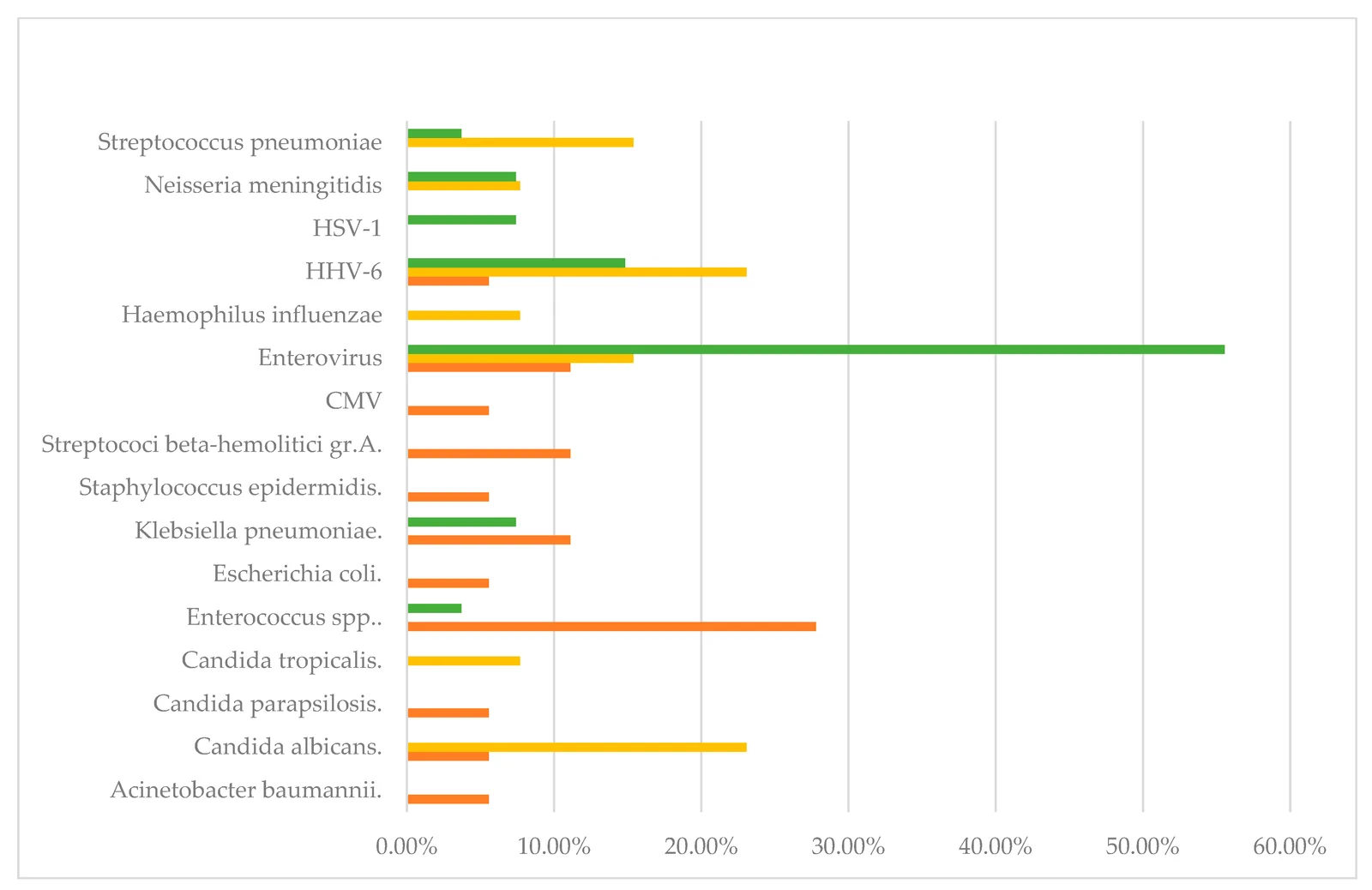
Distribution of identified etiological agents or agent groups among confirmed pediatric meningitis episodes. Data are stratified by year: 2025, green; 2024, yellow; 2023, orange.

The distribution of identified etiological agents or agent groups is shown in Figure 1. Enterovirus was the dominant identified agent in 2025, accounting for 15 of 27 confirmed meningitis episodes (55.56%) and 15 of 21 viral meningitis episodes (71.43%). Across the entire study period, enterovirus represented 19 of 58 confirmed episodes (32.76%) and 19 of 30 viral episodes (63.33%). Classical bacterial meningitis pathogens, including *S. pneumoniae*, *N. meningitidis*, and *H. influenzae*, were still identified, but at lower frequencies. Fungal episodes were represented mainly by *Candida* spp. These agent-level findings are descriptive and should be interpreted as local laboratory-based data.

## 4. Discussion

This retrospective, single-center study describes a changing etiological profile of pediatric meningitis in a tertiary children’s hospital in western Romania. Among 641 suspected episodes investigated between 2023 and 2025, 58 were microbiologically confirmed, yielding an overall positivity rate of 9.05%. Although diagnostic yield remained relatively stable, the distribution of confirmed etiologies shifted toward viral meningitis, particularly in 2025. Overall, 30 episodes were viral, 22 bacterial, and 6 fungal. Viral episodes increased from 22.22% in 2023 to 77.78% in 2025, while bacterial episodes decreased from 66.67% to 22.22%. This pattern is consistent with the conjugate-vaccine and molecular-diagnostic era, in which classical bacterial meningitis has become less frequent and viral etiologies and unresolved aseptic syndromes have become increasingly prominent [6,10,11,12].

In the present cohort, etiological distribution differed significantly according to sex, driven mainly by the predominance of viral meningitis among male patients: 66.67% of male episodes were viral, compared with 35.71% of female episodes. This pattern is consistent with previous reports, including Peer et al. [13]. Conversely, the higher proportion of bacterial and fungal etiologies among episodes involving female patients in our cohort contrasts with larger bacterial meningitis datasets, where male predominance is more commonly reported, including the meta-analysis by Liechti et al. and meningococcal disease data reported by Green et al. [14,15].

Age patterns were also distinct. Bacterial meningitis predominated in infants, accounting for 61.90% of confirmed episodes in the 0–1-year group, whereas viral meningitis predominated after infancy. Viral etiologies represented 78.57% of episodes in children aged 1–6-years and 72.97% of all episodes among children older than 1 year. However, this should not be interpreted as a strict age-based distinction. Infants still require the greatest diagnostic caution because bacterial meningitis carries an immediate risk, while enteroviral disease may closely mimic serious bacterial infection [16,17,18]. Overall, viral disease seems to become proportionally more prominent beyond infancy, without reducing the need for urgent exclusion of bacterial meningitis in younger children.

At the pathogen level, enterovirus was the dominant finding in 2025, accounting for 15 of 27 confirmed meningitis episodes and 15 of 21 viral episodes that year. Across the full study period, enterovirus represented 19 of 58 confirmed episodes and 19 of 30 viral episodes, supporting its central role in pediatric viral meningitis. This is consistent with international data identifying enteroviruses as leading causes of viral and aseptic meningitis in children [6,19,20]. The 2025 increase may reflect local variation in enterovirus circulation. However, given the short study period and lack of genotyping or population-based data, it may be viewed as a possible local surge rather than a sustained trend.

Although bacterial meningitis was less frequent than viral meningitis in this cohort, it remains clinically essential. Classical bacterial pathogens, including *Streptococcus pneumoniae*, *Neisseria meningitidis*, and *Haemophilus influenzae*, were still identified, and bacterial meningitis continues to carry high risks of rapid progression, mortality, neurological sequelae, and urgent public health implications [4,16,21]. Therefore, the microbiology laboratory has a dual role: rapid detection of high-risk bacterial and fungal etiologies requiring immediate targeted management, and confirmation of viral etiologies that may reduce diagnostic uncertainty and support antimicrobial stewardship.

These findings support an integrated diagnostic approach to suspected pediatric meningitis. Conventional methods remain essential for bacterial and fungal confirmation, susceptibility testing, and surveillance, while molecular assays improve the detection of viral agents, particularly enteroviruses and herpesviruses. Rapid PCR can provide early etiological guidance and support treatment de-escalation, but should complement rather than replace conventional microbiology. Negative results must still be interpreted alongside clinical findings, CSF parameters, Gram stain, culture, sampling timing, prior antimicrobial exposure, and local epidemiology [20,22]. In our cohort, only 58 of 641 suspected episodes were microbiologically confirmed, possibly reflecting noninfectious mimics, pretreatment, low pathogen load, limited CSF volume, pathogens outside the panel, or etiologies requiring serological investigation. The findings therefore support structured diagnostic pathways and improved reporting of viral meningitis because of its effects on hospitalization, empirical treatment, laboratory workload, and clinical decision-making [23,24].

Romanian evidence on pediatric meningitis remains limited and has focused mainly on bacterial meningitis, invasive meningococcal disease, or viral outbreaks [25]. The 1999 aseptic meningitis outbreak and the 2012 Suceava enterovirus cluster confirm the epidemiological relevance of viral meningitis, but also show that available data are often historical or outbreak-based rather than derived from routine hospital surveillance [26,27]. More recent national evidence indicates that viral etiologies, particularly enteroviruses, are frequently identified in children, although many suspected episodes remain unconfirmed [28]. Our study addresses this gap by describing pediatric meningitis surveillance in a routine hospital setting that integrates conventional CSF microbiology with molecular diagnostics.

The under-characterization of viral meningitis extends beyond Romania and reflects a broader surveillance problem. Viral meningitis and related CNS infections may be underreported when symptoms are mild, etiological testing is incomplete, or episodes remain classified as aseptic meningitis without pathogen identification [29]. Although prognosis is generally more favorable than in bacterial disease, pediatric episodes still impose substantial clinical and operational burdens through hospitalization, empirical treatment, repeated investigations, parental anxiety, bed occupancy, and laboratory workload [30]. Improved characterization and reporting are therefore important for surveillance, healthcare planning, resource allocation, and antimicrobial stewardship.

### Limitations and Future Perspectives

This study has several limitations. Its retrospective, single-center, laboratory-based design limits generalizability, and the findings should be interpreted as local hospital surveillance data rather than population-level epidemiological evidence. Although 641 suspected episodes were analyzed, only 58 were microbiologically confirmed, limiting subgroup and pathogen-level analyses. Sparse categories, particularly fungal etiologies, included expected cell counts below 5; therefore, these results were interpreted cautiously and checked using exact or Monte Carlo-based contingency-table approaches where applicable.

For each episode, the available variables included patient age group, sex, area of origin, admitting ward, and method of microbiological confirmation. Although such information may have been available and considered within the routine clinical and laboratory diagnostic workflow, detailed clinical variables—including vaccination status, comorbidities, immune status, antibiotic pretreatment, CSF cytological and biochemical parameters, disease severity, treatment, length of hospitalization, complications, and outcomes—were not systematically extracted in a standardized form for all episodes. As a result, clinical predictors, prognostic associations, and the impact of microbiological diagnosis on patient management could not be assessed. Because the same patient could contribute more than one temporally distinct hospitalization, analyses were conducted at the episode level and did not account for within-patient clustering.

Additional limitations relate to case ascertainment and diagnostic workflow. Because only hospitalized children who underwent CSF microbiological testing were included, mild episodes, episodes without lumbar puncture, or episodes not submitted for microbiological investigation were not captured. Molecular testing was performed within routine clinical workflows and was not applied systematically to all suspected meningitis episodes. Its use may have been influenced by clinical indication, available CSF volume, assay availability, and changes in laboratory practice. Consequently, differences in molecular testing coverage or ordering practices between years may have influenced the detection of viral pathogens, particularly the apparent increase observed in 2025.

Despite these limitations, the study provides useful local laboratory surveillance data on the etiological distribution of pediatric meningitis and highlights an observed predominance of viral meningitis among children older than 1 year. Future studies should build on these findings using larger multicenter cohorts, standardized molecular testing algorithms, and more complete clinical datasets. This would allow better assessment of age-specific etiological patterns, seasonality, vaccination status, antibiotic pretreatment, disease severity, treatment decisions, and outcomes. Further research should also clarify the clinical impact of rapid molecular diagnosis, including its role in antimicrobial de-escalation, hospitalization duration, and optimization of diagnostic pathways for suspected pediatric meningitis.

## 5. Conclusions

In this retrospective, single-center laboratory-based study, confirmed pediatric meningitis episodes showed a heterogeneous etiological profile, with a redistribution of confirmed etiologies over time. Although the annual positivity rate remained relatively stable between 2023 and 2025, viral meningitis increased and became the leading etiology overall, while bacterial meningitis decreased proportionally but remained predominant among infants.

Enterovirus accounted for the largest proportion of confirmed episodes in 2025, suggesting an important local contribution of viral meningitis during the study period. Viral etiology was also more frequent among male patients and among older children. This age-related pattern was supported both by the predominance of viral episodes after the first year of life and by the higher median age of viral episodes compared with non-viral episodes, with univariable analysis showing increasing odds of viral etiology with each additional year of age.

These findings support the value of integrating molecular testing with conventional microbiological methods in suspected pediatric meningitis. Rapid molecular assays may improve early etiological orientation, particularly for viral agents, while culture and antimicrobial susceptibility testing remain essential for bacterial and fungal confirmation, targeted therapy, and surveillance. Better characterization and reporting of viral meningitis are therefore relevant for epidemiological surveillance, healthcare planning, laboratory resource allocation, and antimicrobial stewardship.

## Figures and Tables

**Table 1:** Annual distribution of suspected and confirmed pediatric meningitis episodes, 2023–2025.

Year	Suspected Episodes (n)	Negative (n)	Positive (n)	Positivity Rate
2023	160	142	18	11.25%
2024	193	180	13	6.74%
2025	288	261	27	9.38%
Total	641	583	58	9.05%

**Table 2:** Characteristics of microbiologically confirmed meningitis episodes.

Variable	Category	n	%
Total		58	100.00
Age, years	Median, IQR	2.00	0.00–4.75
	Range	0.00–15.00	
Age group	<1 year	21	36.21
	1–6 years	28	48.28
	7–18 years	9	15.52
Sex	Male	30	51.72
	Female	28	48.28
Residence	Urban	38	65.52
	Rural	20	34.48
Admitting department	Emergency Department	24	41.38
	Intensive Care Unit	19	32.76
	Preterm Neonatal Ward	11	18.97
	Neonatal Pathology Ward	2	3.45
	Preterm Neonatal Intensive Care Unit	2	3.45

**Table 3:** Annual etiological distribution of confirmed pediatric meningitis episodes.

Etiology	2023 (n, %)	2024 (n, %)	2025 (n, %)	Total (n, %)
Bacterial	12 (66.67%)	4 (30.77%)	6 (22.22%)	22 (37.93%)
Fungal	2 (11.11%)	4 (30.77%)	0 (0.00%)	6 (10.34%)
Viral	4 (22.22%)	5 (38.46%)	21 (77.78%)	30 (51.72%)
Total	18	13	27	58

**Table 4:** Etiological variation based on sex.

Etiology	Female, n (%)	Male, n (%)	Total, n (%)
Bacterial	13/28 (46.43%)	9/30 (30.00%)	22/58 (37.93%)
Fungal	5/28 (17.86%)	1/30 (3.33%)	6/58 (10.34%)
Viral	10/28 (35.71%)	20/30 (66.67%)	30/58 (51.72%)
Total	28/58 (48.28%)	30/58 (51.72%)	58/58 (100%)

**Table 5:** Etiological distribution of confirmed pediatric meningitis episodes by age group.

Etiology	<1 Year (n, %)	1–6 Years (n, %)	7–18 Years (n, %)	Total (n, %)
Bacterial	13 (61.90%)	6 (21.43%)	3 (33.33%)	22 (37.93%)
Fungal	5 (23.81%)	0 (0.00%)	1 (11.11%)	6 (10.34%)
Viral	3 (14.29%)	22 (78.57%)	5 (55.56%)	30 (51.72%)
Total	21	28	9	58

**Table 6:** Age distribution according to viral versus non-viral etiology and exploratory association between age and viral meningitis.

Analysis	Group/Predictor	n	Median Age, Years	IQR	Effect Estimate (95% CI)	*p*-Value
Descriptive comparison	Viral etiology	30	4	2–5	—	<0.001 *
	Non-viral etiology	28	0	0–2	—	
Univariable logistic regression	Age, per 1-year increase	58	—	—	OR = 1.22 (1.01–1.48)	0.041 ^†^

* Mann–Whitney U test comparing viral and non-viral episodes. ^†^ Univariable logistic regression; outcome: viral etiology versus non-viral etiology.

## Data Availability

Data are available upon request from the corresponding author.
